# Supplementary material for: Extensive flavivirus E trimer breathing accompanies stem zippering of the post‐fusion hairpin
Source: EMBO Rep. 2020 Jun 2;21(8):e50069. doi: 10.15252/embr.202050069 (PMC7403712; doi:10.15252/embr.202050069)
Supplement: Supplementary file 1 — Appendix [file EMBR-21-e50069-s001.pdf]

Appendix

Extensive flavivirus E trimer breathing accompanies stem zippering of the post-fusion hairpin

Iris Medits‡, Marie-Christine Vaney‡, Alexander Rouvinski†, Martial Rey, Julia Chamot-Rooke, Felix A. Rey*, Franz X. Heinz*, Karin Stiasny*

‡ These authors contributed equally to this work

* Corresponding authors:
karin.stiasny@meduniwien.ac.at
franz.x.heinz@meduniwien.ac.at
felix.rey@pasteur.fr

The Appendix file includes:

Page 2. Appendix Figure S1. Multiple sequence alignment of flavivirus prM and E.
Page 4. Appendix Figure S2. Crystal packing of the sE-linker* trimer.
Page 5. Appendix Figure S3. Characterization of oligomeric states of TBEV (s)E proteins.
Page 6. Appendix Table S1. X-ray data collection and refinement statistics.
Page 7. Appendix Table S2. Statistical analyses of thermostability data shown in Figure 4B.
Page 8. Appendix Table S3. Statistical analyses of mab binding data shown in Figure 6B.

Appendix Figure S1

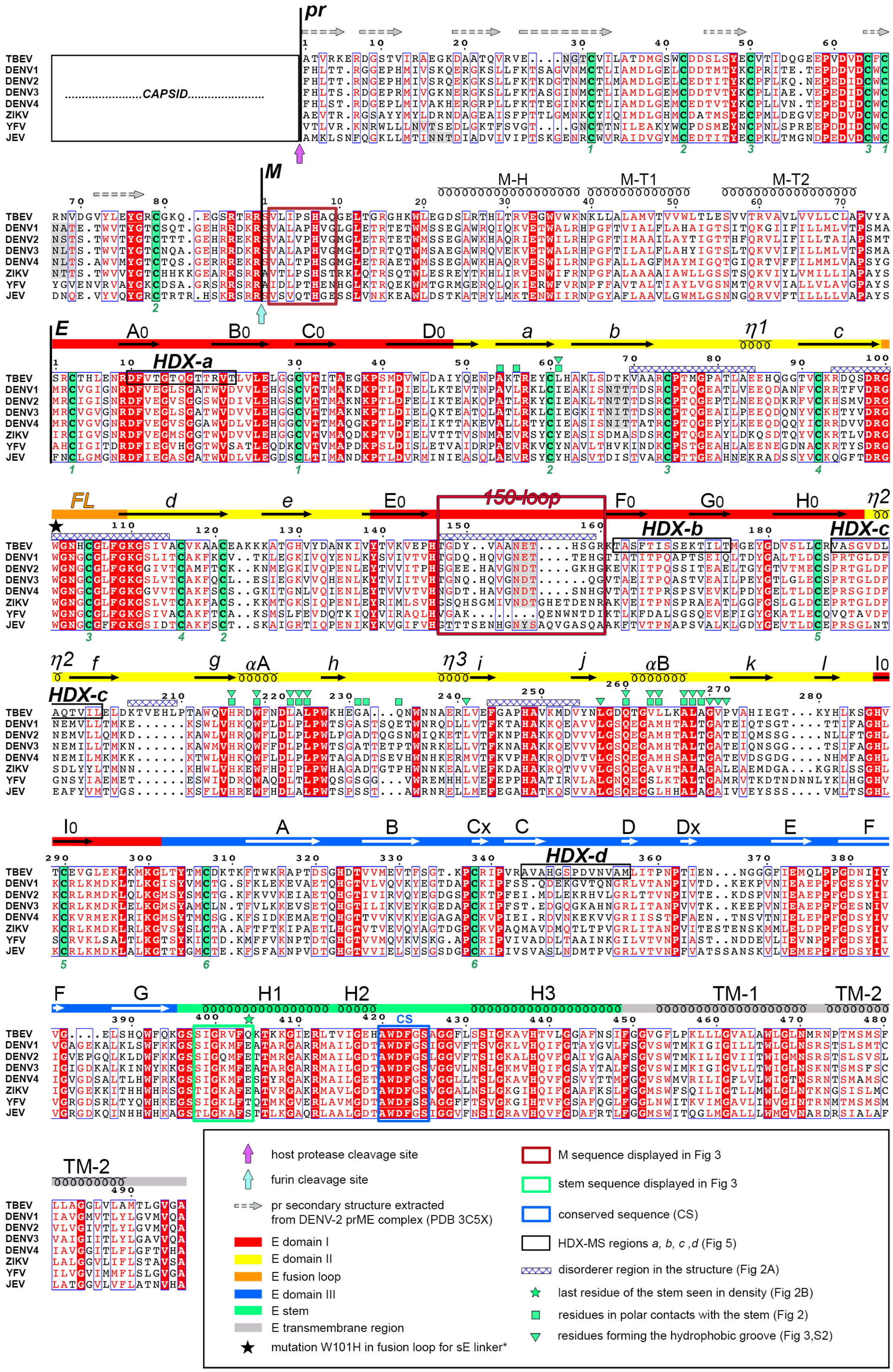

Appendix Figure S1. Multiple sequence alignment of flavivirus prM and E.

Positions that are strictly identical are shown in white font on a red background, and positions with residues with similar properties are in red font. Conserved cysteines are highlighted on a green background, with a green number below the alignment identifying their disulfide partner. A bar colored according to the E domains over the portion corresponding to E indicates the secondary structure elements observed in the sE-linker* structure. The pr secondary structure (dashed arrows in grey) was extrapolated from that of pr in the X-ray structure of the DENV2 pr/sE heterodimer (PDB 3C5X). The M secondary structure is from the mature virion (PDB 5O6A). Purple and cyan vertical arrows mark the host signal peptidase and furin cleavage sites, respectively. The key below defines additional symbols used on the alignment. This Figure was generated with the Espript 3.0 web server (http://espript.ibcp.fr) [66].

Appendix Figure S2

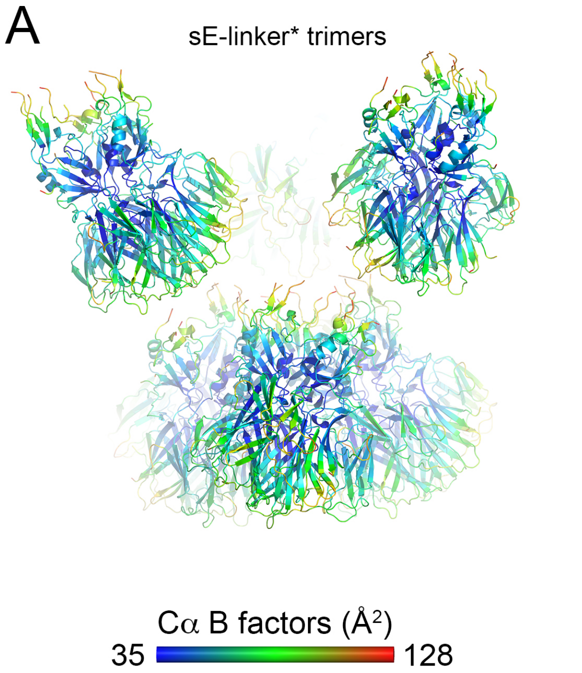

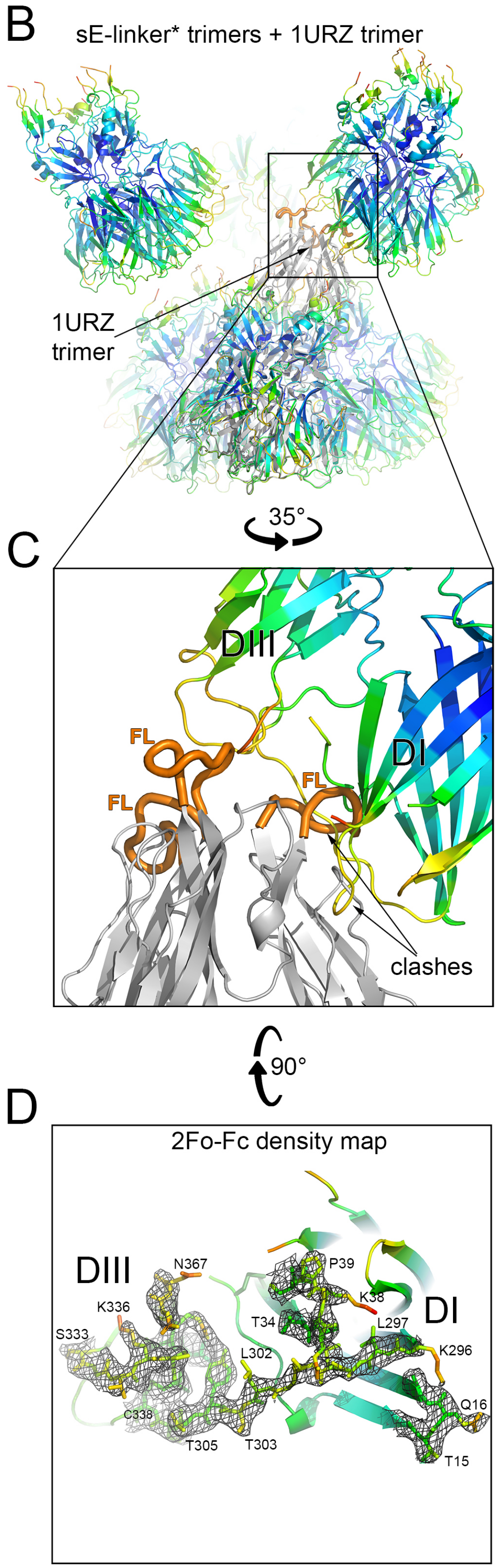

Appendix Figure S2. Crystal packing of the TBEV sE-linker* trimer.

A. Trimers in contact in the crystal, colored according to the crystallographic temperature B-factor of alpha carbon atoms as in the color bar key.

B. Same view as in (A) but with one TBEV sE trimer from the sE401v structure (PDB 1URZ) in which the domain II-tip is ordered (shown in grey with the FLs in orange), superposed on one sE-linker* trimer in the crystal.

C. Close-in of the framed region in (B), rotated by 35 degrees, of one sE 1URZ trimer (in grey with FLs in orange) and one sE-linker* trimer (colored according to temperature factors). The domain II-tip of the full trimer clashes against the base of the sE-linker* trimer (domains I and III).

D. View at 90 degrees to that in (C), showing that residues in the linker between domains I and III and nearby loops, which would have clashed with the fusion loop, are indeed fully ordered in the crystal. These residues were built into the final model using the final 2Fo-Fc electron density map (in black mesh) and fit well into their density, with the exception of some disordered side chains.

Appendix Figure S3

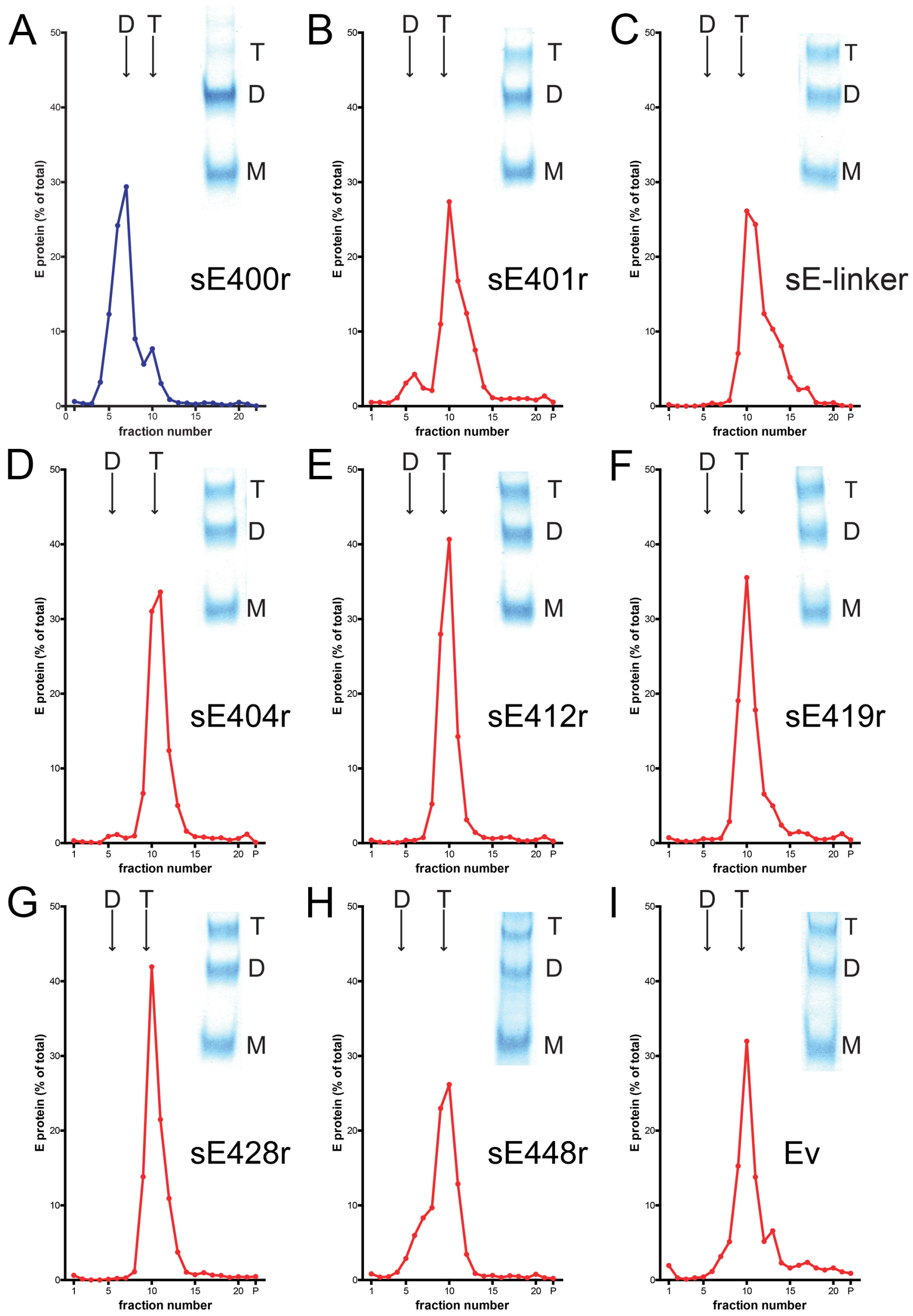

Appendix Figure S3. Characterization of oligomeric states of TBEV (s)E proteins.

A. sE400r preparation.

B-I. Trimers used in this study.

Sedimentation analyses of protein preparations subjected to conditions that induce trimerization. The amount of E protein in each fraction was determined by a quantitative four-layer ELISA as described in Materials and Methods.

Sedimentation is from left to right. Inset: SDS-PAGE after chemical crosslinking with dimethylsuberimidate. Position of monomers (M), dimers (D) and trimers (T) are indicated.

Appendix Table S1.
X-ray data collection and refinement statistics.

	sE-linker*<br>PDB 6S8C	sE401v<br>PDB 1URZ
Crystallization conditions		
	100mM Na citrate pH 5.6<br>19% (w/v) PEG 4000<br>19% isopropanol<br>5% glycerol	100mM Na acetate pH 4.5<br>25% (w/v) PEG 4000<br>0.3% (w/v) DDAO[£]
Data Collection [\$]		
Space group	H 3	
Cell dimensions		
a, b, c (Å)	169.4, 169.4, 123.6	
α, β, γ (°)	90.0, 90.0, 120.0	
Resolution (Å)	48.9-2.57 (2.67-2.57)	
R*merge (%)*	9.9 (98.0)	
R*meas (%)*	13.5 (1.3)	
R*pim (%)*	7.2 (73.1)	
< I / σ(I) >	7.9 (1.1)	
$CC_{1/2}$ (%)	99.4 (29.0)	
Measured reflections	147146 (15174)	
Unique reflections	41959 (4356)	
Completeness (%)	99.5 (97.5)	
Multiplicity	3.5 (3.5)	
Refinement [\$]		
Resolution	48.9-2.57 (2.64-2.57)	
N° of Work / Free reflections	41941 / 2028 (3014 / 139)	
R*work* / R*free* (%)	21.5 / 23.0 (23.1 / 23.5)	
N° of protein atoms / waters	7770 / 85	
R.m.s. deviations		
Bond lengths (Å)	0.007	
Bond angles (°)	0.93	
Ramachandran plot		
Favored (%)	95.2	
Allowed (%)	4.7	
Outliers (%)	0.1	

[£] DDAO, N,N-dimethyl decylamine oxide

[\$] Highest resolution shell is shown in parenthesis.

$R_{merge} = \Sigma_h\Sigma_i(|<I_h> - I_{h,i}|)/\Sigma_h\Sigma_i I_{h,i}$ (where h are the unique reflections and i their symmetry-equivalent)
$R_{work} = \Sigma|F_o - F_c|/\Sigma|F_o|$; $R_{free} = \Sigma|F_o - F_c|/\Sigma|F_o|$ using 5% of the F_o selected randomly.

< I / σ(I) >, empirical signal-to-noise ratio; $CC_{1/2}$, correlation coefficient; R.m.s., root mean square; R*meas*, multiplicity-corrected R*merge*; R*pim*, expected precision of merged data.

Appendix Table S2.
Statistical analyses of thermostability data shown in Figure 4B.

	**Ev** *n=6	**sE401v** *n=5	**sE401r** *n=3	**sE404r** *n=5	**sE412r** *n=4	**sE419r** *n=6	**sE428r** *n=3	**sE448r** *n=3	**sE-linker** *n=4
**Ev**		****	****	ns	ns	ns	ns	ns	****
p value	-	<0.0001	<0.0001	0.29	0.8413	0.7279	0.7301	0.9887	<0.0001
**sE401v**	****		ns	****	****	****	****	****	*
p value	<0.0001	-	0.9941	<0.0001	<0.0001	<0.0001	<0.0001	<0.0001	0.0104
**sE401r**	****	ns		****	****	****	****	****	**
p value	<0.0001	0.9941	-	<0.0001	<0.0001	<0.0001	<0.0001	<0.0001	0.0045
**sE404r**	ns	****	****		ns	**	*	ns	****
p value	0.29	<0.0001	<0.0001	-	0.997	0.0076	0.0208	0.1171	<0.0001
**sE412r**	ns	****	****	ns		ns	ns	ns	****
p value	0.8413	<0.0001	<0.0001	0.997	-	0.0937	0.1369	0.4641	<0.0001
**sE419r**	ns	****	****	**	ns		ns	ns	****
p value	0.7279	<0.0001	<0.0001	0.0076	0.0937	-	>0,9999	>0,9999	<0.0001
**sE428r**	ns	****	****	*	ns	ns		ns	****
p value	0.7301	<0.0001	<0.0001	0.0208	0.1369	>0.9999	-	0.9987	<0.0001
**sE448r**	ns	****	****	ns	ns	ns	ns		****
p value	0.9887	<0.0001	<0.0001	0.1171	0.4641	>0.9999	0.9987	-	<0.0001
**sE-linker**	****	*	**	****	****	****	****	****	
p value	<0.0001	0.0104	0.0045	<0.0001	<0.0001	<0.0001	<0.0001	<0.0001	-

* biological replicates ns, not significant; ****, p<0.0001; ***,p<0.001; **,p<0.01; *,p<0.05

*biological replicates

ns, not significant; ****,p<0.0001; ***,p<0.001; **,p<0.01; *,p<0.05

Data were analyzed by ANOVA and Tukey´s multiple-comparison test

Appendix Table S3.
Statistical analyses of mab binding data shown in Figure 6B.

**A1 reactivity**						
	**sE401v** *n=4	**sE404r** *n=4	**sE419r** *n=4	**sE428r** *n=5	**sE448r** *n=4	**Ev** *n=4
**sE401v**	-	ns	ns	****	****	****
p value				<0,0001	<0,0001	<0,0001
**sE404r**	ns	-	ns	****	****	****
p value				<0,0001	<0,0001	<0,0001

**A2 reactivity**						
	**sE401v** *n=4	**sE404r** *n=4	**sE419r** *n=4	**sE428r** *n=4	**sE448r** *n=3	**Ev** *n=4
**sE401v**	-	ns	***	****	****	****
p value			<0,0001	<0,0001	<0,0001	<0,0001
**sE404r**	ns	-	****	****	****	****
p value			<0,0001	<0,0001	<0,0001	<0,0001

**A3 reactivity**						
	**sE401v** *n=4	**sE404r** *n=4	**sE419r** *n=4	**sE428r** *n=4	**sE448r** *n=4	**Ev** *n=4
**sE401v**	-	ns	**	****	**	*
p value			0.0076	<0,0001	0.0037	0.0256
**sE404r**	ns	-	ns	***	*	ns
p value				0.0006	0.0335	

**B4 reactivity**						
	**sE401v** *n=4	**sE404r** *n=4	**sE419r** *n=6	**sE428r** *n=3	**sE448r** *n=4	**Ev** *n=4
**sE401v**	-	ns	**	***	****	*
p value			0.0018	0.0001	<0,0001	0.0121
**sE404r**	ns	-	***	****	****	**
p value			0.0008	<0,0001	<0,0001	0.0063

*biological replicates

ns, not significant; ****,p<0.0001; ***,p<0.001; **,p<0.01; *,p<0.05

Data were analyzed by ANOVA and Dunnett´s multiple-comparison test
