# Supplementary material for: Extensive flavivirus E trimer breathing accompanies stem zippering of the post‐fusion hairpin
Source: EMBO Rep. 2020 Jun 2;21(8):e50069. doi: 10.15252/embr.202050069 (PMC7403712; doi:10.15252/embr.202050069)
Supplement: Supplementary file 2 — Expanded View figures PDF [file EMBR-21-e50069-s002.pdf]

Expanded View Figures

Figure EV1. The αA/B hydrophobic groove.

A Simulated annealing omit map contoured at 1σ values showing clear density for the stem in the sE-linker* structure.

B, C The αA/B groove in the post-fusion E trimer (B) and in the pre-fusion E dimer of mature virions (C) is essentially hydrophobic. All four panels show a surface representation color-coded by hydrophobicity following the color bar underneath. (B, left) TBEV E C-ter residues 399–403 interacting in the groove (Gln404 of the stem was omitted for clarity, as it interacts with the adjacent subunit, see Fig 2B). Right, same view but with the surface rendered semi-transparent to show the hydrophobic side chains (property conserved across flaviviruses) lining the groove. Strictly conserved residues are underlined. (C, left) TBEV M N-ter residues 2–8 interacting in the groove (see Fig 3A). Right, same view on a semi-transparent surface as above.

 © 2020 The Authors

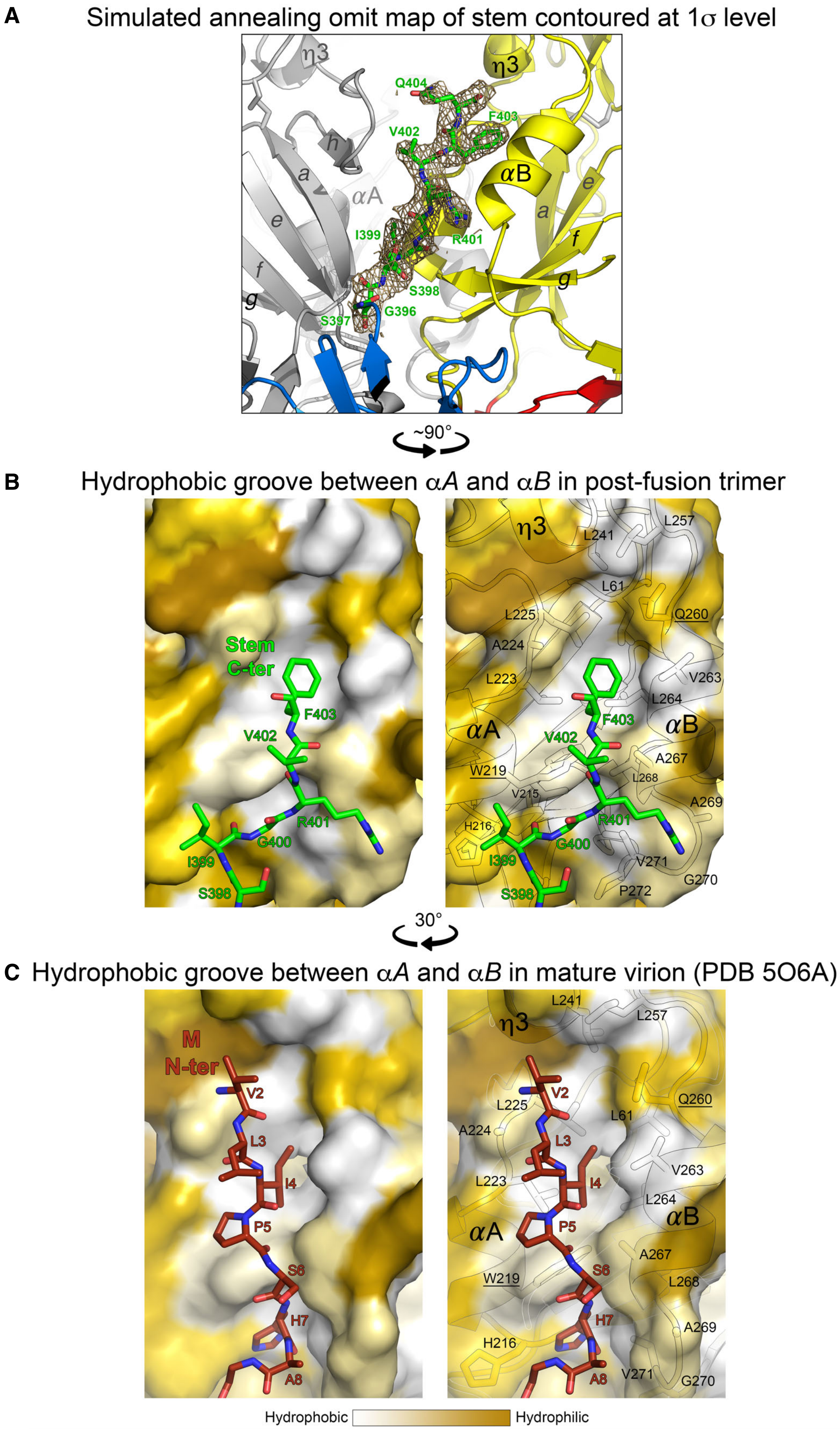
A
Simulated annealing omit map of stem contoured at 1σ level
η3
η3
Q404
F403
V402
αB
αA
I399
R401
S398
G396
S397
~90°
B
Hydrophobic groove between αA and αB in post-fusion trimer
Stem C-ter
F403
V402
R401
G400
I399
S398
L257
L241
L61
L225
Q260
A224
L223
V263
L264
αA
αB
A267
W219
L268
V215
A269
H216
V271
G270
P272
30°
C Hydrophobic groove between αA and αB in mature virion (PDB 5O6A)
M N-ter
V2
L3
I4
P5
S6
H7
A8
L225
A224
L223
L61
Q260
V263
L264
A267
L268
A269
H216
V271
G270
Hydrophobic
Hydrophilic

Figure EV1.

Figure EV2. The αA/B groove and its interactions in flaviviruses of known structure.

The αA/B groove (left column) and two close-ups (middle and right columns) showing the side chains in interaction in the cryo-EM structures of mature flavivirus virions: top panel, DENV2 (PDB 3J2P); middle panel, ZIKV (PDB 6CO8) and bottom panel, JEV (PDB 5WSN). M is shown in brown and E colored according to domains. In the top panel, the X-ray structure of the closely related DENV1 sE trimer (PDB 4GSX) was superposed on helices αA and αB, to show the path of the stem (green) within the groove. The tryptophan residue (W219 in TBEV numbering) is strictly conserved and thus is the hydrogen bond its side chain makes with the main-chain carbonyl of residue 5 in M (brown dashed lines).

 © 2020 The Authors

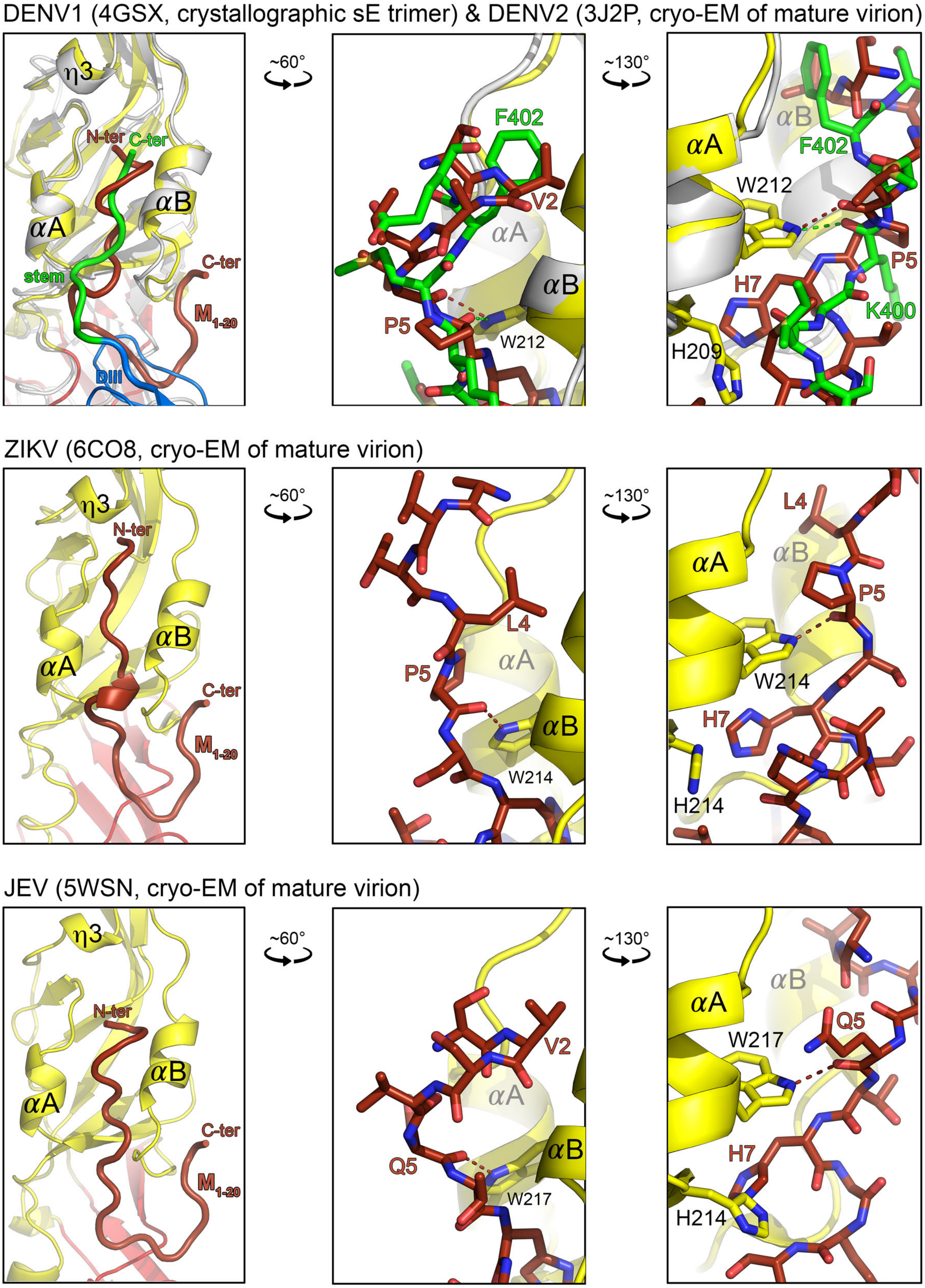
DENV1 (4GSX, crystallographic sE trimer) & DENV2 (3J2P, cryo-EM of mature virion)
η3
N-ter
C-ter
αA
αB
stem
C-ter
M1-20
DIII
~60°
F402
V2
αA
αB
P5
W212
~130°
αB
αA
F402
W212
P5
H7
K400
H209
ZIKV (6CO8, cryo-EM of mature virion)
η3
N-ter
αB
αA
C-ter
M1-20
~60°
L4
P5
αA
αB
W214
~130°
L4
αA
αB
P5
W214
H7
H214
JEV (5WSN, cryo-EM of mature virion)
η3
N-ter
αB
αA
C-ter
M1-20
~60°
V2
αA
αB
Q5
W217
~130°
αB
αA
Q5
W217
H7
H214

Figure EV2.

Figure EV3. Hydrogen–deuterium exchange mass spectrometry.

Difference in deuterium uptake between constructs sE404r and sE419r (D_{404}–D_{419}) (top), of sE404r and sE428r (D_{404}–D_{428}) (middle) and of sE419r and sE428r (D_{419}–D_{428}) (bottom). The top panel corresponds to panel (A) shown in Fig 5, displayed here next to the results obtained when comparing the exchange difference in 404–428 to highlight the very similar pattern observed in the two cases (top and middle). The exchange difference between sE419r and sE428r shows only small differences in deuterium uptake (bottom). The same scale and threshold were applied to the three plots.

 © 2020 The Authors

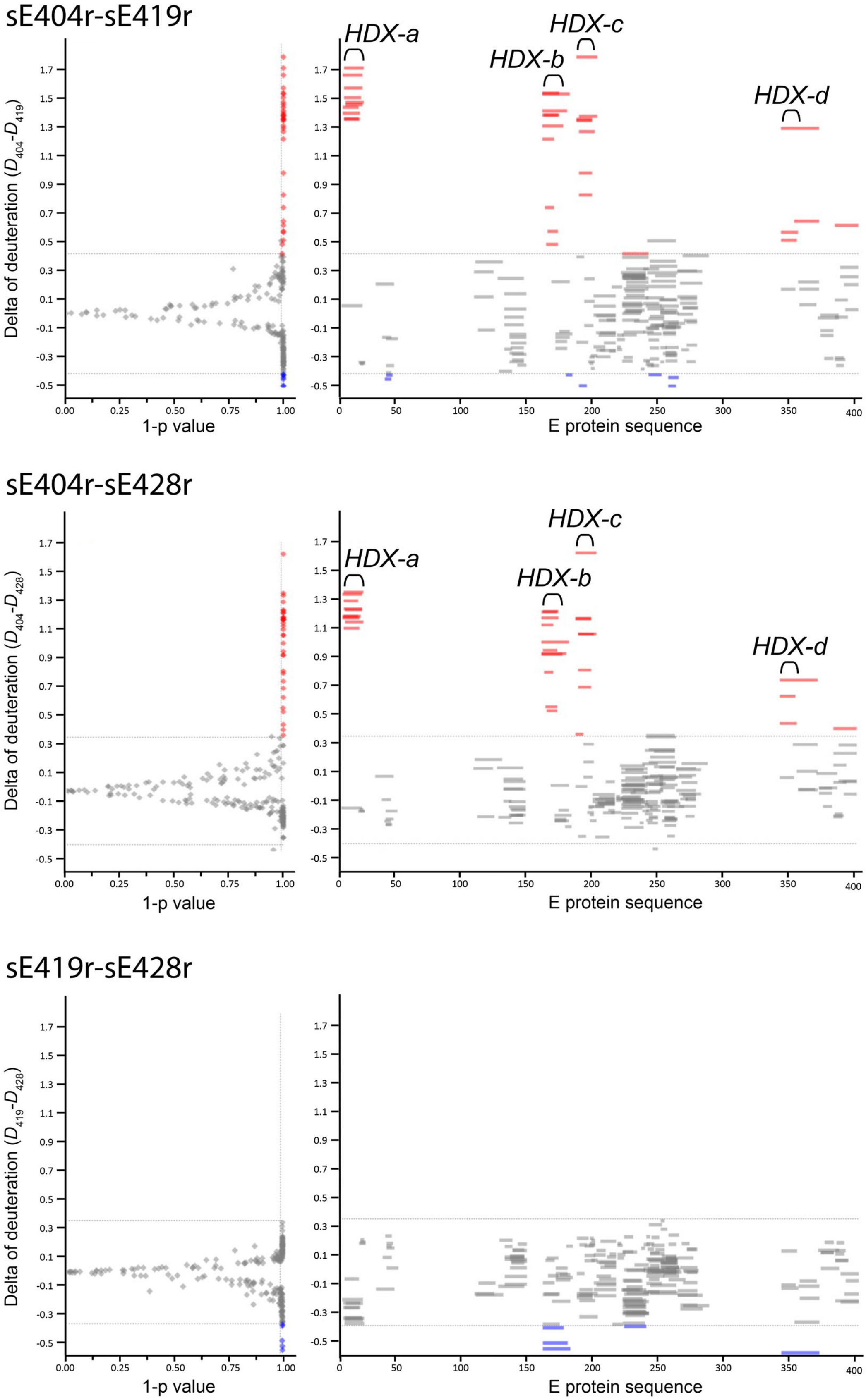
sE404r-sE419r
Delta of deuteration (D_{404}-D_{419})
1-p value
E protein sequence
HDX-a
HDX-b
HDX-c
HDX-d
sE404r-sE428r
Delta of deuteration (D_{404}-D_{428})
1-p value
E protein sequence
HDX-a
HDX-b
HDX-c
HDX-d
sE419r-sE428r
Delta of deuteration (D_{419}-D_{428})
1-p value
E protein sequence

Figure EV3.

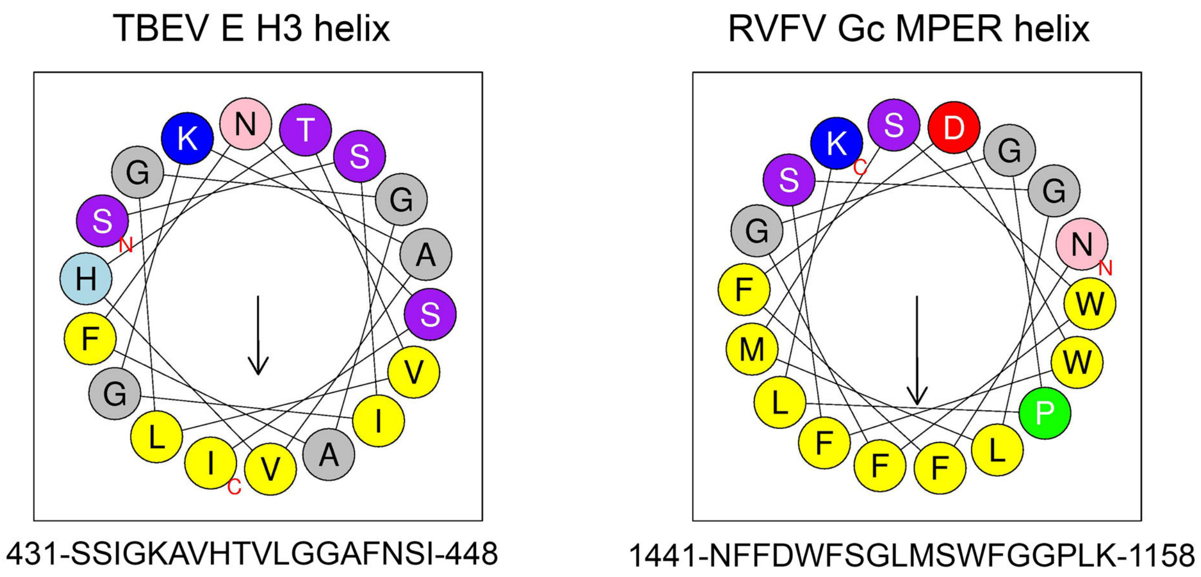

Figure EV4. Membrane-proximal external region (related to Fig 7).

Helical wheel representation of the sequence segment spanning the H3 region of TBEV E and that of the MPER of RVFV Gc, located between the end of the stem zipper at the level of the FL and the viral TM region. The helical wheel diagrams were made with the HELIQUEST server [63]. The amino acids are shown in circles colored according to residue character: non-polar (yellow), alanine, glycine (gray, small or no side chain), proline (green, introduces a kink), and polar (all other colors). The arrow in the helical wheels corresponds to the hydrophobic moment (index of the amphiphilicity of a helix). The N- and C-terminal ends are indicated in small red fonts. The corresponding aa sequences are indicated below the wheel.

 © 2020 The Authors
