# Supplementary material for: Extensive flavivirus E trimer breathing accompanies stem zippering of the post‐fusion hairpin
Source: EMBO Rep. 2020 Jun 2;21(8):e50069. doi: 10.15252/embr.202050069 (PMC7403712; doi:10.15252/embr.202050069)
Supplement: Supplementary file 4 — Source Data for Figure 4 [file EMBR-21-e50069-s003.pdf]

Source data Figure 4B

| % of trimer at 37°C (a) | | | | | | | | |
|---|---|---|---|---|---|---|---|---|
| **Ev** | **sE 401v** | **sE 401r** | **sE 404r** | **sE 412r** | **sE 419r** | **sE 428r** | **sE 448r** | **sE-linker** |
| 90.70 | 28.03 | 13.84 | 65.83 | 67.98 | 100.00 | 90.10 | 89.76 | 39.00 |
| 69.00 | 22.53 | 26.05 | 65.70 | 67.39 | 82.40 | 88.03 | 79.12 | 34.79 |
| 83.12 | 18.31 | 27.75 | 72.30 | 81.59 | 78.77 | 99.23 | 92.87 | 49.31 |
| 88.76 | 26.89 | | 56.77 | 73.65 | 86.50 | | | 38.40 |
| 76.92 | 30.56 | | 81.82 | | 100.00 | | | |
| 79.01 | | | | | 95.19 | | | |

(a) Trimer stability expressed as percentage of the fraction detected in the trimer peak in sedimentation analyses after incubation at 70°C relative to 37°C (100%)

| Ev set to 100% (b) | | | | | | | | |
|---|---|---|---|---|---|---|---|---|
| **Ev** | **sE 401v** | **sE 401r** | **sE 404r** | **sE 412r** | **sE 419r** | **sE 428r** | **sE 448r** | **sE-linker** |
| 100 | 35.48 | 17.52 | 83.32 | 86.04 | 126.57 | 114.04 | 113.61 | 49.36 |
| | 28.52 | 32.97 | 83.16 | 85.30 | 104.29 | 111.42 | 100.14 | 44.03 |
| | 23.18 | 35.12 | 91.51 | 103.27 | 99.70 | 125.60 | 117.55 | 62.41 |
| | 34.03 | | 71.85 | 93.22 | 109.48 | | | 48.60 |
| | 38.68 | | 103.56 | | 126.57 | | | |
| | | | | | 120.48 | | | |

(b) Data shown in Fig 4B, normalized with respect to the corresponding Ev fraction
